# *In vitro* mouse preantral follicle development in 2D and suspension culture: α-MEM vs SAGE 1-step

**DOI:** 10.1530/RAF-25-0158

**Published:** 2026-07-22

**Authors:** Amir Bazgir, Narges Karami, Fatemeh Hassani, Farah Farokhi, Azam Dalman

**Affiliations:** ^1^ Department of Biology, Faculty of Science, Urmia University, Urmia, Iran; ^2^ Department of Stem Cells and Developmental Biology, Faculty of Basic Sciences and Advanced Technologies in Biology, University of Science and Culture, Tehran, Iran; ^3^ Department of Embryology, Reproductive Biomedicine Research Center, Royan Institute for Reproductive Biomedicine, ACECR, Tehran, Iran

**Keywords:** folliculogenesis, *in vitro* maturation (IVM), oocyte maturation, culture media, three-dimensional (3D) culture, mouse ovarian follicle

## Abstract

**Abstract:**

The *in vitro* culture of immature follicles remains a challenge in reproductive biology, ART, and fertility preservation. This study investigated the impact of α-MEM and SAGE 1-Step media on mouse preantral follicle development and oocyte maturation using two-dimensional (2D) and suspension culture systems. Preantral follicles (∼130 μm) from 14-day-old NMRI mice were cultured for 13 days in either medium under 2D or suspension conditions. On day 13, hCG was added to induce meiotic resumption. We evaluated follicular growth, antrum formation, oocyte maturation, meiotic spindle organization, and expression of genes related to maturation (*Bmp15, Gdf9*), cumulus expansion (*Has2, Ptgs2, Lhr, Adamts1*), and apoptosis (*Bax, Bcl2*). Antral formation was higher in α-MEM in both culture systems (*P* > 0.05). However, SAGE 1-Step significantly improved oocyte maturation across both systems (*P* = 0.01 in 2D vs *P* = 0.006 in suspension compared to α-MEM). Spindle staining demonstrated that suspension culture significantly increased the percentage of oocytes with normal meiotic spindle organization (*P* = 0.002). Gene expression analysis revealed a significant upregulation of *Adamts1* (*P* = 0.008) and downregulation of *Bax* (*P* = 0.01) in suspension culture. Furthermore, the apoptotic ratio of *Bax*/*Bcl2* was significantly decreased in the suspension system (*P* = 0.01). In conclusion, the SAGE 1-Step medium significantly enhances oocyte maturation, while the suspension system superiorly preserves meiotic spindle architecture and reduces apoptosis. The combination of SAGE 1-Step and suspension culture provides an optimized microenvironment for producing high-quality oocytes.

**Lay summary:**

Growing immature egg outside the body, a process known as *in vitro* follicle culture, is a promising but challenging technique for preserving fertility. This study investigated novel approaches to improve this process, aiming to create healthier, more viable eggs for future use. The researchers found that using a specific medium significantly improved egg development and maturation. Importantly, they found that culturing these immature eggs in suspension, where the follicles remained floating in the medium, rather than in a traditional two-dimensional system where follicles inevitably flatten and partially lose their three-dimensional structure, resulted in significantly better outcomes. Eggs grown in suspension showed more organized cellular structures and reduced cell death compared to those cultured on a flat surface. These findings represent an important step toward reliably producing healthy, developmentally competent eggs outside the body, offering new hope for fertility preservation and assisted reproduction.

## Introduction

*In vitro* maturation (IVM) of ovarian follicles is a vital technique for elucidating the mechanisms of folliculogenesis and offers a promising fertility preservation option for patients facing gonadotoxic treatments (Quan *et al.* 2020, Telfer & Andersen 2021, Malo *et al.* 2024, Vitale & Dolmans 2024). However, achieving high rates of competent oocytes from *in vitro* cultured preantral follicles remains challenging, as *in vitro*-derived oocytes often display reduced developmental competence compared to naturally derived ones (Eppig & O’Brien 1996). Therefore, optimizing culture conditions is crucial.

Initially, two-dimensional (2D) systems were widely used; however, in these systems, the follicular structure is inevitably compromised, leading to flattening and disruption of the follicular architecture. This impairs the essential intercellular interactions, ultimately leading to reduced oocyte competence and epigenetic instability (Jensen & Teng 2020, Gargus & Woodruff 2021, Dey *et al.* 2025, Luciano *et al.* 2025). Attention shifted to three-dimensional (3D) environments, which better maintain spatial architecture, improve hormonal responsiveness, and support gene expression profiles that closely resemble native follicles (Belli *et al.* 2012, Desai *et al.* 2012). While scaffold-based 3D systems (e.g. using hydrogels such as alginate or PEG) recreate an *in vivo*-like microenvironment, they present challenges such as difficult oocyte retrieval and variable degradation rates (He 2017, Dadashzadeh *et al.* 2021, Xiang *et al.* 2021).

Consequently, scaffold-free 3D cultures, such as those using ultra-low attachment (ULA) plates, have become popular for preserving spherical morphology (Ren *et al.* 2022, Converse *et al.* 2023, Zisiadi *et al.* 2025). In this study, we applied a matrix-free suspension culture using suspension Petri dishes and microdroplet systems instead of ULA plates. This novel configuration allows for easier pipetting and manipulation of follicles and oocytes while successfully maintaining 3D architecture and critical intercellular signaling, thereby enhancing experimental feasibility.

Alongside physical support, the biochemical composition of the medium is critical. α-Minimal essential medium (α-MEM) is a standard baseline that supports preantral follicle morphology but often yields relatively low oocyte maturation rates (Jimenez *et al.* 2016, Abouzaripour *et al.* 2018).

Alternatively, SAGE 1-Step is a continuous culture medium widely used in human *in vitro* fertilization (IVF) (Fesahat *et al.* 2017, López-Pelayo *et al.* 2018, Al-Hafedh *et al.* 2024). Unlike traditional sequential media, continuous single-step media, such as SAGE 1-Step, are formulated with stable forms of glutamine (e.g. L-alanyl-L-glutamine) and robust antioxidants. This composition minimizes metabolic stress, prevents fluctuations in temperature and pH during handling, and protects developing cells against oxidative stress over prolonged culture periods (Chronopoulou & Harper 2015).

Given the necessity of optimizing follicle culture protocols, this study aimed to comprehensively investigate and compare the *in vitro* development of mouse preantral follicles and oocyte maturation using SAGE 1-Step versus α-MEM under both 2D and suspension culture systems. The evaluation focused on follicle morphology, developmental progression, meiotic spindle organization, and the expression of genes critical for oocyte competence and apoptosis.

## Materials and methods

### Experimental design

Mouse preantral follicles were cultured in either α-MEM (Gibco, USA) or SAGE 1-Step (Origio, Denmark) medium, each supplemented with 5% FBS (Gibco, USA), 100 mIU/mL FSH (Gonal-F, Germany), 50 μg/mL ascorbic acid (Sigma-Aldrich, Germany), and 1% ITS (insulin, transferrin, and selenium; Gibco, USA). To evaluate these conditions concurrently, experiments were conducted simultaneously using a 2 × 2 factorial design, where both culture media were tested across both 2D and suspension culture systems. Follicles in all four experimental groups were maintained for 13 days. On day 13, 5 IU/mL hCG (Merck, Germany) was added to induce meiosis and oocyte maturation. Antrum formation and oocyte maturation rates were assessed using an inverted microscope. After the optimal culture medium was identified, oocyte quality was evaluated by immunocytochemical staining of meiotic spindles. Gene expression related to maturation (*Bmp15, Gdf9*), cumulus expansion (*Has2, Ptgs2, Lhr, Adamts1*), and apoptosis (*Bax, Bcl2* and the *Bax/Bcl2* ratio) was then compared between the 2D and suspension culture systems using quantitative real-time PCR (qRT-PCR).

### Ethical approval

This study was approved by the Ethics Committee of the Royan Research Institute (Approval Number: IR.ACECR.AEC.1401.022).

### Preantral follicle isolation and culture

Ovaries were collected from 14-day-old NMRI mice maintained in a temperature-controlled facility (20–25°C) under a 12 h light:12 h darkness cycle. After dissection, the ovaries were transferred to droplets of α-MEM culture medium supplemented with 10% FBS, pre-warmed in an incubator for 4–6 h. Preantral follicles ranging from 120 to 130 μm in diameter were selected and isolated. Following washing, follicles were randomly assigned to experimental groups and cultured for 13 days either in a 2D system using tissue culture-treated Petri dishes (430166, Corning, USA) or in a suspension system using suspension culture dishes (430589, Corning, USA, Supplementary Fig. 1 (see section on Supplementary materials given at the end of the article)). In both systems, follicles were individually cultured in 20 μL microdroplets of the respective media covered with mineral oil (Sigma-Aldrich, Germany). Every 2–3 days, half of the medium volume (10 μL) was carefully removed and replaced with fresh medium to replenish essential nutrients, maintain optimal pH, and remove metabolic waste without causing excessive stress to the developing follicles (Hassani *et al.* 2019). The follicles were cultured for a total of 13 days (Hassani *et al.* 2019, Jamalzaei *et al.* 2020, Amjad *et al.* 2024). Accordingly, on day 13, follicles that had developed a visible antral cavity were selected for maturation and transferred to fresh medium containing the same supplements, along with 5 IU/mL hCG. After 18–21 h of incubation (on day 14), oocytes were released from their surrounding cumulus cells by gentle pipetting.

### Evaluation of follicle development rate and oocyte maturation

Follicle morphology was assessed on days 0, 6, 9, and 13 of culture. Formal morphological assessments were initiated from day 6 onward, as the initial period (days 1–4) primarily represents an adaptation phase to the *in vitro* environment, during which morphological divergence among the experimental groups is minimal. On day 13, follicle survival and antrum formation rates were determined in viable follicles. Follicles that appeared darkened, had prematurely lost their oocyte, or exhibited growth arrest (defined as a failure to increase in follicular diameter combined with morphological signs of apoptosis or atresia over consecutive observations) were classified as degenerated. Follicles displaying a distinct antral cavity within the granulosa cell layer were identified as antral follicles. To assess the maturation stage on day 14, cumulus cells were removed by gentle pipetting and, when required, by brief enzymatic digestion using hyaluronidase (85 IU, Sigma-Aldrich, Germany). Accordingly, the stained oocytes were fully denuded and did not contain cumulus cells, which allowed clear evaluation of their maturation status. Oocytes were examined under an inverted microscope to evaluate nuclear maturation based on the presence or absence of the first polar body. Oocytes containing a visible germinal vesicle were classified as GV (germinal vesicle) oocytes. Those lacking both a germinal vesicle and a polar body were identified as GVBD (germinal vesicle breakdown) oocytes, while those containing a polar body were classified as mature MII oocytes. Oocytes that appeared darkened or morphologically abnormal were considered degenerated. The percentage of retrieved oocytes was calculated as follows:

(No. of cumulus–oocyte complexes (COCs) recovered/No. of antral follicles) × 100 = % Oocytes retrieved.

### Meiotic spindle immunostaining

Meiotic spindle immunostaining was performed to assess spindle structure and chromosome alignment at the metaphase plate. Oocytes from each group were fixed in 4% paraformaldehyde for 30 min, followed by washes in PBS (Gibco, USA) containing 0.01% Tween 20 (Gibco, USA). Cell membranes were permeabilized with Triton X-100 for 15 min to allow antibody penetration. Non-specific binding was blocked by incubation in PBS containing 0.3% goat serum (S-26, Sigma-Aldrich, Germany) for 1 h at 37°C. Oocytes were incubated with a FITC-conjugated anti-alpha-tubulin primary antibody (ab64503, Abcam, UK, 1:150 dilution) for 1 h at 37°C. After PBS-T washes, nuclei were counterstained with 1 μg/mL 4′,6-diamidino-2-phenylindole (DAPI; Gibco, USA) for 5 min. Samples were examined under a fluorescence microscope (Olympus, Japan). A normal MII spindle was defined as a barrel-shaped structure with chromosomes tightly aligned at the metaphase plate equator. Spindles were classified as disorganized if they exhibited elongated, multipolar, or collapsed microtubule structures. Chromosomes were considered misaligned if they were dispersed or scattered away from the metaphase plate. The experiment was performed in three independent experiments, and a minimum of 10 oocytes were analyzed per group.

### qRT-PCR analysis

Total RNA was extracted from follicles (day 14: 15–20 preovulatory follicles per replicate) using the RNeasy Micro Kit (Qiagen, USA). Complementary DNA (cDNA) was synthesized according to the manufacturer’s instructions using the SMOBio cDNA Synthesis Kit (SMOBio, USA). The resulting cDNA was amplified with gene-specific oligonucleotides and SYBR Green (Thermo Fisher, USA). The primer sequences are listed in Table 1. Gene expression analysis focused on *Gdf9, Bmp15, Bax, Bcl2, Has2, Ptgs2, Lhr,* and *Adamts1*, with *Gapdh* used as the housekeeping gene. Additionally, the *Bax/Bcl2* ratio, a well-established indicator of apoptosis, was calculated from the relative expression levels of these genes. For gene expression analysis, samples were collected from five independent experiments per group, and each PCR was run with two technical replicates. A uniform fluorescence threshold and identical baseline settings were applied across all samples within each assay. Fold changes were calculated relative to the 2D group (as the calibrator) using the 2^−ΔΔCt^ method.

**Table 1:** Primer information.

Gene	Primer pair (5′–3′)		Product length (bp)
	Forward	Reverse	
*Gdf9*	CAA​ACC​CAG​CAG​AAG​TCA​C	AAG​AGG​CAG​AGT​TGT​TCA​GAG	194
*Bmp15*	AAATGGTGAGGCTGGTAA	TGAAGTTGATGGCGGTAA	148
*Bax*	GAG​AGG​TCT​TTT​TCC​GAG​TGG​C	TGT​CCC​AAA​GTA​GGA​GAG​GAG	237
*Bcl2*	CCA​CTT​AGG​ACC​CAC​TTC​TGA​C	GGG​TGC​TTC​CTA​CAG​CTA​CAG​T	188
*Has2*	CCT​CAT​CAT​CCA​AAG​CCT​G	ACA​TTT​CCG​CAA​ATA​GTC​TG	138
*Ptgs2*	AGG​AGG​TCT​TTG​GTC​TGG​TG	TCT​GGA​ACA​ACT​GCT​CAT​CG	126
*Lhr*	CTG GCC TAG CCA CCG GAG CTC	CTG GGG CGC CCT GTA CTC ACA G	157
*Adamts1*	AAG​AAG​TTT​GAT​AAG​TGT​GGC​GT	CCC​TTT​GAT​TCC​GAT​GTT​TCA​C	154

### Statistical analysis

Data distribution was first assessed for normality using the Kolmogorov–Smirnov test. Antral cavity formation and rates of maturation were analyzed using two-way ANOVA followed by Tukey’s post hoc test across four independent experiments. Furthermore, proportional data in the text are presented in the format of ‘(*n*/total), %’ to ensure transparency. Abnormal oocyte data (from three independent experiments) and gene expression levels (from five independent experiments) were analyzed using an independent-samples *t*-test. To provide precise statistical reporting, exact *P*-values are documented for all comparisons rather than standard inequalities, with *P* < 0.05 considered statistically significant.

## Results

### Comparison of α-MEM and SAGE 1-Step media on preantral follicle development, morphology, and oocyte maturation in 2D and suspension systems

**Figure 1:**
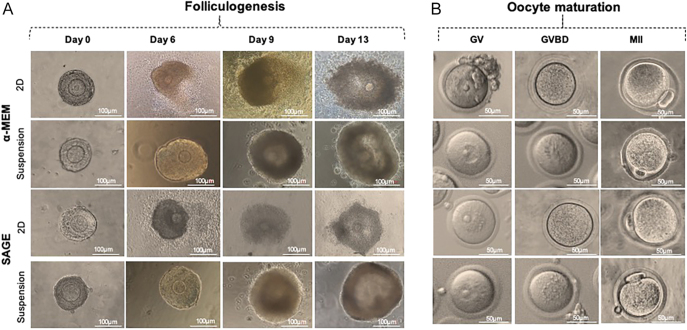
Morphological assessment of *in vitro* follicle development and subsequent oocyte maturation. (A) Bright-field images of follicle development over 13 days (days 0, 6, 9, 13) in α-MEM and SAGE 1-Step media under 2D and suspension systems. Note the follicle flattening in the 2D system compared to the retained spherical architecture in suspension. (B) Representative images of retrieved oocytes in the germinal vesicle (GV), germinal vesicle breakdown (GVBD), and metaphase II (MII) stages. Scale bars: 100 μm (follicles) and 50 μm (oocytes). All images are representative of four independent experiments.

Morphological assessment revealed that follicles cultured in the 2D system underwent inevitable flattening and loss of their three-dimensional architecture, regardless of the medium used. In contrast, the suspension system successfully preserved the spherical structure of the follicles throughout the 13-day culture period (Fig. 1). In the suspension culture, as follicles increased in size and weight, some settled at the bottom of the droplets, establishing weak, late-stage attachments to the dish surface. This resulted in a qualitative observation of localized granulosa cell migration; however, the follicles effectively retained their overall 3D integrity, appearing more cohesive compared to those in the 2D system. Furthermore, follicles and oocytes cultured in the SAGE 1-Step medium exhibited a more natural and healthier appearance compared to those in α-MEM (Fig. 1).

**Figure 2:**
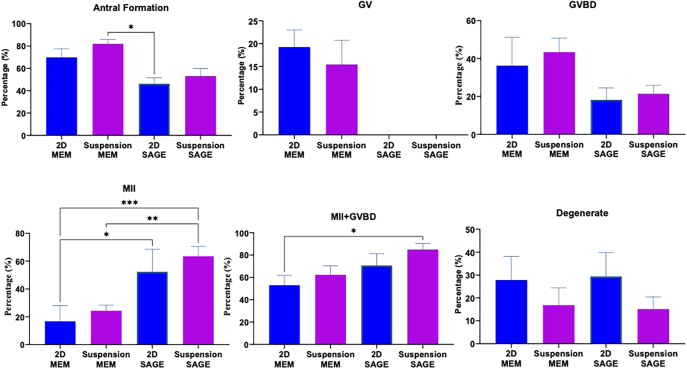
Quantitative analysis of *in vitro* follicle development, meiotic maturation, and degeneration rates. The bar charts illustrate the percentage of antrum formation, rates of oocyte meiotic maturation (arrested at GV, progressing to GVBD, and reaching MII or GVBD + MII), and the percentage of degenerated oocytes across the culture groups. Data are presented as mean percentages ± SEM (four independent experiments). Statistical analysis was performed using two-way ANOVA with Tukey’s post hoc test, following arcsine transformation of the percentage data. Asterisks indicate statistically significant differences between groups (*P = 0.01, **P = 0.006, ***P = 0.0006).

Regarding developmental rates, the transition to the antral stage was numerically lower in the SAGE 1-Step medium than in α-MEM in both 2D (46.25 ± 5.1%, (31/70) vs 69.82 ± 7.88%, (46/68); *P* = 0.22) and suspension conditions (53.05 ± 6.8%, (36/68) vs 81.96 ± 4.06%, (57/70); *P* = 0.07, Fig. 2). Despite this, the oocyte maturation rate (MII stage) was significantly improved in the SAGE 1-Step medium across both culture systems (52.38 ± 16.03%, (15/31) vs 16.69 ± 11.27%, (7/46) in 2D, *P* = 0.01, and 63.46 ± 7.01%, (22/36) vs 24.35 ± 3.94%, (14/57) in suspension, *P* = 0.006; Fig. 2).

When comparing culture systems, the suspension system yielded higher rates of antrum formation and oocyte maturation (GVBD and MII) compared to the 2D system, although these differences did not reach statistical significance (*P* > 0.05). Additionally, a non-significant reduction in oocyte degeneration was observed in the suspension system across both media compared to 2D cultures (16.86 ± 7.5%, (10/57) vs 27.78 ± 10.27%, (13/46) in α-MEM, *P* = 0.9, and 15.09 ± 5.40%, (6/36) vs 29.36 ± 10.47%, (10/31) in SAGE 1-Step, *P* = 0.7; Fig. 2).

Overall, the SAGE 1-Step medium outperformed α-MEM in supporting oocyte meiotic resumption and maturation. Based on these findings, SAGE 1-Step was selected as the optimal medium for subsequent experiments. To further evaluate the impact of the culture system, oocyte spindle quality and the expression of key genes related to folliculogenesis and apoptosis were compared between 2D and suspension systems using the SAGE 1-Step medium.

### Evaluation of mature oocyte quality by immunocytochemistry

**Figure 3:**
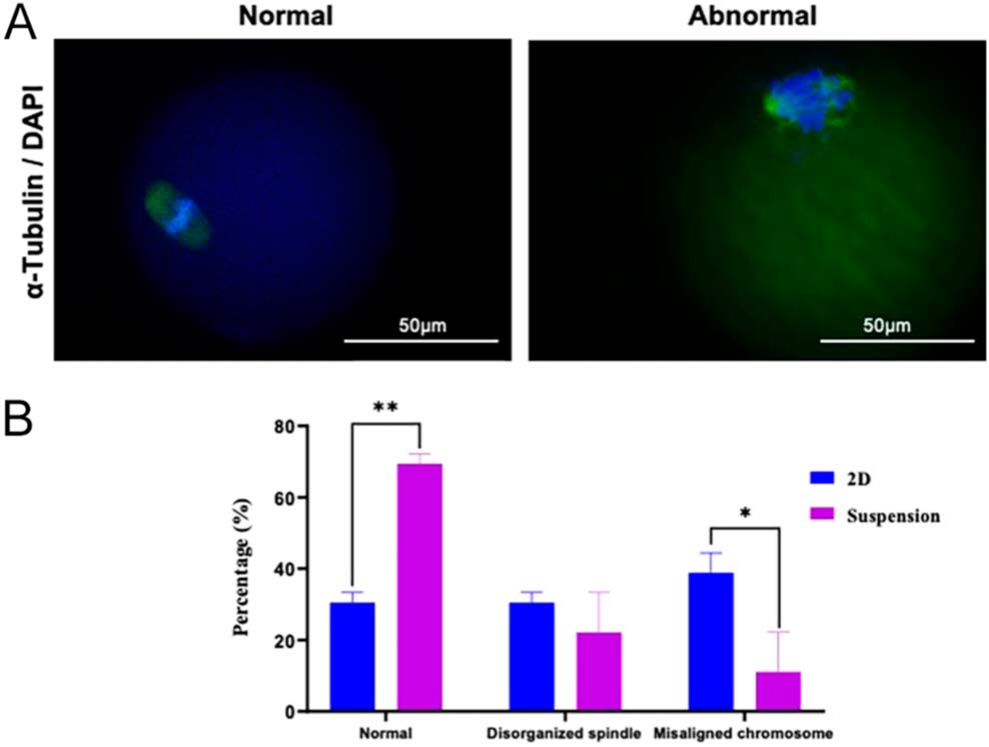
Immunofluorescence staining and quantitative analysis of α-tubulin organization in *in vitro*-matured oocytes cultured in the SAGE 1-Step medium under 2D and suspension systems. (A) Fluorescence images of meiotic spindle morphologies. ‘Normal’ indicates a well-organized barrel-shaped spindle, whereas ‘Abnormal’ shows a disorganized spindle and misaligned chromosomes (green: α-tubulin/FITC; blue: chromosomes/DAPI). Scale bar: 50 μm. (B) Percentage of oocytes with normal versus abnormal α-tubulin morphology. Data are mean ± SEM from three independent experiments (10 oocytes/group). Statistical significance was determined using an independent *t*-test (*P = 0.01, **P = 0.002).

Suboptimal culture conditions may impair coordinated nuclear and cytoplasmic maturation, leading to spindle disorganization and chromosomal misalignment, which ultimately compromise embryo developmental potential (Moor *et al.* 1998). As shown in Fig. 3, the proportion of oocytes with normal spindle organization and chromosome alignment at the metaphase plate was higher in the SAGE-1 step medium with suspension system (69.44 ± 2.77%, (7/10)) compared with the 2D system (30.55 ± 2.77%, (3/10); *P* = 0.002). Correspondingly, the rate of chromosomal misalignment was significantly lower in the suspension group (11.11 ± 11.11%, (1/10) than in the 2D system (38.88 ± 5.55%, (4/10); *P* = 0.01; Fig. 3).

### Gene expression evaluation

**Figure 4:**
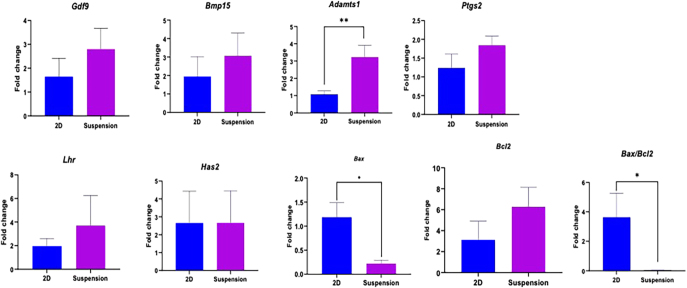
Relative gene expression related to folliculogenesis and apoptosis in 2D versus suspension cultures (SAGE 1-Step medium) on day 14. The graphs compare relative mRNA levels (fold change calculated via the 2^−ΔΔCt^ method) of *Gdf9*, *Bmp15*, *Ptgs2*, *Bcl2*, *Adamts1*, *Has2, Lhr*, *Bax*, and the *Bax/Bcl2* ratio between 2D (blue bars) and suspension (violet bars) systems. Statistical analysis was performed using an independent *t*-test (*P = 0.01, **P = 0.008).

The expression of eight genes associated with folliculogenesis (*Gdf9, Bmp15, Has2, Ptgs2, Lhr,* and *Adamts1*) and apoptosis (*Bax* and *Bcl2*) was analyzed (Fig. 4). The expression levels of *Gdf9, Bmp15, Ptgs2, Lhr*, and *Bcl2* tended to be higher in the suspension culture system compared to the 2D group, although the difference is not significant. In contrast, *Has2* expression did not differ between systems. Notably, *Adamts1* expression was significantly upregulated in the suspension system (*P* = 0.008), whereas the pro-apoptotic gene *Bax* was significantly increased in the 2D culture system (*P* = 0.01). Consequently, the *Bax/Bcl2* expression ratio was significantly higher in the 2D system compared to the suspension culture (*P* = 0.01), indicating a stronger apoptotic drive in the 2D configuration (raw data for Fig. 4 are available in Supplementary File 1).

## Discussion

Follicles represent the fundamental structural and functional units of the female reproductive system, and their culture condition optimization *in vitro* remains a cornerstone of fertility preservation strategies. In this study, we compared the effects of two culture media, α-MEM and SAGE 1-Step, on the *in vitro* development of mouse preantral follicles in both 2D and suspension systems. Our findings highlight that both the biochemical composition of the culture medium and the physical configuration of the culture system are critical determinants of follicular survival, antrum formation, oocyte maturation, and gene expression profiles.

Recent research has underscored the pivotal role of biomaterial scaffolds in recreating the ovarian niche to enhance *in vitro* follicular development. Systems incorporating Wharton’s jelly or mesenteric peritoneal matrix have demonstrated improved follicular viability, antral formation, and angiogenic activity (Sarabadani *et al.* 2021, Zand *et al.* 2023), whereas amniotic membrane-based hydrogels exhibited favorable biocompatibility with limited impact on maturation (Haghshenas *et al.* 2022). Concordant with the goals of these complex scaffold-based approaches, our findings indicate that the simple, scaffold-free suspension system, when combined with an optimized culture medium (SAGE 1-Step), can similarly promote superior oocyte maturation and quality without the need for exogenous matrices. Collectively, these data reinforce the concept that integrating biochemical composition with appropriate physical architecture is essential for successful folliculogenesis under *in vitro* conditions.

Consistent with previous reports (Wang *et al.* 2013, Filatov *et al.* 2020), α-MEM supported early follicular growth and progression to the antral stage. As a nutrient-rich basal medium with abundant glucose, α-MEM strongly promotes the proliferation of somatic granulosa cells, which is essential for antrum formation. However, standard basal media, such as α-MEM, typically contain L-glutamine, which spontaneously degrades at 37^∘^C into ammonia during prolonged culture – a by-product known to be highly embryotoxic and negatively affect developmental competence after oocyte activation (Kim *et al.* 2013).

In contrast, the enriched SAGE 1-Step medium, originally designed for embryo culture, significantly enhanced *in vitro* oocyte maturation. This superior performance is directly linked to its specialized composition (Farsi *et al.* 2013, Fesahat *et al.* 2017), which closely mimics the physiological requirements of the maturing oocyte. Unlike α-MEM, SAGE 1-Step incorporates stable dipeptides of glutamine, which prevent the accumulation of toxic ammonia over the prolonged 13-day culture period (Chronopoulou & Harper 2015). Furthermore, SAGE 1-Step addresses the specific metabolic shift required by the maturing oocyte. While granulosa cells metabolize glucose, the oocyte itself relies heavily on pyruvate and lactate for energy. The inclusion of calcium lactate in SAGE 1-Step directly supplies this preferred energy substrate, promoting mitochondrial activity, supporting cytoplasmic maturation, and enhancing overall oocyte competence (Kim *et al.* 2011).

Additionally, SAGE 1-Step is supplemented with hyaluronan, a major macromolecule in follicular fluid. Its presence enhances gap junction communication between cumulus cells and the oocyte, facilitating the transfer of regulatory molecules essential for cumulus expansion and nuclear maturation (Opiela *et al.* 2014).

These findings suggest that optimal *in vitro* follicle development may benefit from a staged culture strategy: α-MEM could be utilized during the initial growth phase to maximize antrum formation, followed by a transition to the SAGE 1-Step or SAGE IVM medium to fulfill the specific metabolic and energetic demands of final oocyte maturation.

In the suspension culture system, follicles not only retained their natural spherical structure but also preserved intercellular communications, particularly between the oocyte and surrounding granulosa cells. This study employed a droplet culture approach within suspension Petri dishes, which not only facilitated handling but also maintained the follicle’s three-dimensional architecture. When combined with the SAGE 1-Step medium, this system yielded higher rates of oocyte maturation, fewer oocytes arrested at the GV stage, and a lower proportion of degenerated oocytes. By contrast, the 2D system disrupted the native follicular structure in both media, likely due to surface adhesion and mechanical stress, ultimately compromising oocyte quality. These findings reinforce previous studies highlighting the critical role of preserving the follicle’s three-dimensional integrity in achieving successful oocyte maturation (Desai *et al.* 2010, Khunmanee & Park 2022).

In terms of nuclear and cytoplasmic maturation, the suspension culture system more effectively supported the natural organization of meiotic spindles and proper chromosome alignment at the metaphase plate when cultured in the SAGE 1-Step medium. By contrast, oocytes derived from 2D culture frequently exhibited spindle disorganization and chromosome misalignment. These findings indicate that suspension culture promotes not only nuclear but also cytoplasmic maturation, thereby enhancing the developmental competence of the oocyte and potentially improving the quality of the resulting embryos (Combelles *et al.* 2002).

For the first time, this study evaluated the expression of genes related to oocyte maturation, cumulus cell expansion, and apoptosis in preovulatory follicles cultured in the SAGE 1-Step medium under 2D and suspension conditions. Two critical oocyte-secreted factors, *Gdf9* and *Bmp15*, which regulate cumulus cell proliferation, differentiation, and metabolism (Chang *et al.* 2016), were numerically higher in the suspension system, although the difference did not reach statistical significance due to data variability. Previous research has highlighted the essential role of GDF9 and BMP15 in fertility, with deficiencies in animal models resulting in severe defects in folliculogenesis and infertility (Bodin *et al.* 2007). As highlighted by Gilchrist *et al.* (2008), optimal paracrine signaling mediated by these oocyte-secreted factors is crucial for coordinating granulosa cell function and ensuring the production of developmentally competent oocytes.

In addition, downstream pathways including *HAS2, PTGS2, *and* GREM1* in cumulus cells are known to regulate oocyte maturation and competence (McKenzie *et al.* 2004, Bhardwaj *et al.* 2016). Of these, *Has2* and *Ptgs2*, both regulated by *Gdf9* and associated with embryo quality through their role in cumulus cell differentiation and expansion (Wathlet *et al.* 2011, Uyar *et al.* 2013), exhibited similar expression in both groups. Importantly, *PTGS2* is indispensable for cumulus expansion, and its absence results in incomplete maturation of COCs (Lim *et al.* 1997, Faizal *et al.* 2024).

The expression of *Adamts1*, a metalloproteinase essential for ovulation (Curry 2010), was significantly upregulated in the suspension culture, suggesting an enhanced capacity for ovulation and fertilization. *Adamts1* facilitates successful ovulation by selectively degrading components of the extracellular matrix (Shindo *et al.* 2000, Russell *et al.* 2003). Finally, while *Lhr* expression, an established marker of cumulus cell function, is regulated by oocyte-derived factors (Diaz *et al.* 2007), it did not differ significantly between culture conditions; its presence emphasizes the preservation of normal cumulus–oocyte communication in both systems.

Regarding apoptotic pathways, the suspension culture system supported cellular survival by downregulating the pro-apoptotic gene *Bax* while maintaining the expression of the anti-apoptotic gene *Bcl2*. This shift in the *Bax/Bcl2* balance indicates reduced activation of cell death signaling and improved follicular viability. Together, these findings suggest that suspension culture in the SAGE 1-Step medium not only promotes follicle development but also preserves cell integrity, thereby creating a more favorable environment for oocyte maturation.

While this study provides valuable insights into follicle development in the suspension culture system with the SAGE 1-Step medium, it has certain limitations. The developmental competence of the retrieved oocytes, specifically their ability to undergo fertilization, embryo formation, and subsequent development, was not assessed. Additionally, variability in gene expression data limited the statistical significance of certain findings. To strengthen these observations, future studies should be conducted with larger sample sizes and include functional assessments of fertilization potential and embryonic development of the *in vitro*-matured oocytes.

Overall, the findings of this study demonstrate that culturing mouse preantral follicles in the enriched SAGE 1-Step medium within a three-dimensional suspension system effectively supports follicular growth while preserving their natural architecture. This strategy enhances nuclear and cytoplasmic maturation, promotes the expression of key functional genes, and reduces apoptosis, thereby yielding high-quality mature oocytes. Taken together, these results suggest that the suspension culture system combined with the SAGE 1-Step medium represents a promising approach for advancing fertility preservation and may hold potential for future clinical applications.

## Supplementary materials





## Declaration of interest

The authors declare that there is no conflict of interest that could be perceived as prejudicing the impartiality of the work reported.

## Funding

This research did not receive any specific grant from any funding agency in the public, commercial, or not-for-profit sector.

## Author contribution statement

AB contributed to sample preparation, data collection, analysis, and drafting and revising the manuscript. NK contributed to drafting and revising the manuscript. FH contributed to manuscript revision. FF provided overall supervision. AD contributed to the conception and design of the study, manuscript editing, and final approval. All authors read and approved the final manuscript.
