# Supplementary figures and images for: In vitro mouse preantral follicle development in 2D and suspension culture: α-MEM vs SAGE 1-step
Source: Reprod Fertil. 2026 Jul 22;7(3):RAF250158. doi: 10.1530/RAF-25-0158 (PMC13393307; doi:10.1530/RAF-25-0158)

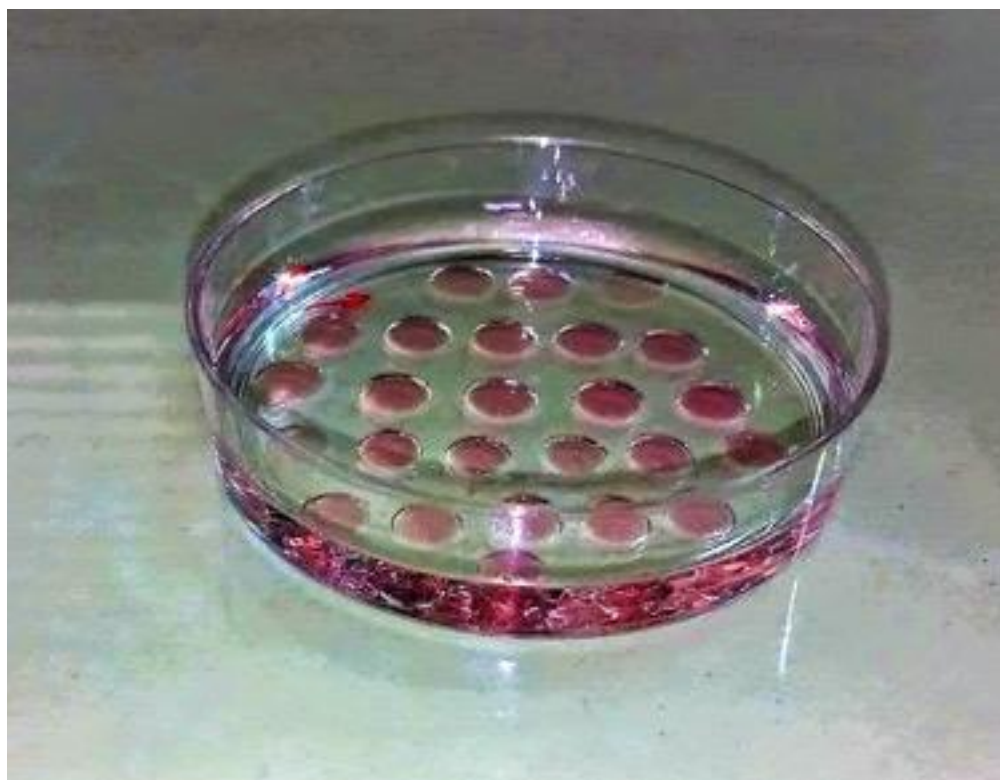

Supplement: Supplementary file 1 [file RAF-25-0158_supplementary_figure_1.pdf]
